# Cost-effectiveness and cost-utility analyses of three different gargles in the treatment of chronic periodontitis

**DOI:** 10.1371/journal.pone.0302592

**Published:** 2024-05-08

**Authors:** Xin Chen, Wenzhi Niu, Guangyu Hu, Changfeng Chen

**Affiliations:** Department of Jiangsu, Xuzhou Stomatological Hospital, Xuzhou, China; UAE University: United Arab Emirates University, UNITED ARAB EMIRATES

## Abstract

**Objective:**

This study aimed to investigate the economics of three different gargles in the treatment of chronic periodontitis.

**Methods:**

A total of 108 patients with periodontitis received one of the following three gargles: xipayi, compound chlorhexidine, or Kangfuxin gargle. The basic information of the patients, the costs of the gargles, the periodontal indexes before and after treatment, and the scores of the 3-level version of the EuroQol Five Dimensions Questionnaire were collected. The cost-effectiveness and cost-utility of the various gargles were determined.

**Results:**

The cost-effectiveness ratios (CER) of the three groups after treatment were 1828.75, 1573.34, and 1876.92 RMB, respectively. The utility values before treatment were 0.92, 0.90, and 0.91, respectively, and the utility values after treatment were 0.98, 0.98, and 0.97, respectively. The cost-utility ratios (CURs) were 213.43, 195.61, and 301.53 RMB, respectively.

**Conclusions:**

For each increase in effective rate and quality-adjusted life years, the treatment cost of periodontitis patients was lower than the gross domestic product per capita of Jiangsu Province, indicating that the treatment cost is completely worth it. The CER and CUR results were the same, and the compound chlorhexidine group was the lowest, demonstrating that when the same therapeutic effect was achieved, it cost the least.

## Introduction

Periodontitis is a type of periodontal tissue damage caused by specific bacterial pathogens and destructive immune responses of the body, which can even lead to the pathological loss of periodontal ligaments and alveolar bone. Periodontitis, like other oral diseases, does not cause death on its own. However, its clear association with known noncommunicable diseases such as diabetes and cardiovascular diseases increases its role in the disease burden of these systemic diseases that can lead to death [1–3]. According to statistical analyses, more than 700 million people (11% of the total population) in the world suffer from severe periodontitis, which has become a widespread disease worldwide and has a serious adverse impact on people’s quality of life and health [4].

Periodontal disease is an inflammatory disease caused by bacterial plaques adhering to the surface of teeth in contact with the gums. The lesions contained in the gingival margin are called gingivitis. Gingivitis can be seen as the result of an immune response and as an attempt by the organism to prevent the development of tissue-destructive components of periodontal disease, known as periodontitis. Periodontitis is characterized by the loss of periodontal alveolar bone and fibers, which eventually leads to the loss of the affected tooth. The course and rate of disease progression are influenced by environmental and genetic factors, including changes in the number of bacteria and their composition in plaques, as well as some medical diseases and medications that affect the immune system. In addition, lifestyle factors, such as tobacco smoking and dental cleaning habits, appear to play an important role in determining disease susceptibility and the rate of progression. Due to its chronic characteristics and the fact that the bacteria that cause the most common forms of periodontitis are endogenous microbes in the human oral flora, periodontitis tends to relapse after treatment [4–6]. Systematic treatment for periodontitis includes the collection of comprehensive information about the patient’s personal oral hygiene, the disease, contributing factors (smoking, etc.) and causes, as well as professional removal of supragingival and subgingival plaques, dental calculus, and overhanging on restorations and crowns [7, 8].

The initiation factors of periodontitis are bacteria, plaque, and its metabolites, so controlling or removing dental plaque plays a very important role in the treatment of periodontitis. At present, the most effective drugs for the clinical control of dental plaque in China are gargles. Xipayi gingival liquid, compound chlorhexidine gargle, and Kangfuxin liquid are common oral gargles used in clinical practice. Several studies have shown that these three kinds of gargle liquids have a significant effect on improving periodontitis and can effectively reduce periodontitis indicators [9–11]. However, there are few economic studies on periodontitis treatments, and in those that have been conducted, their effectiveness is often limited to a single country and its social and economic situation, and some studies only estimated the time required to perform various periodontal treatment procedures without evaluating the economics of their treatment [12–15]. The costs of the three mouthwashes used in clinical practice in China are quite different. Therefore, in this study, we used cost-effectiveness and cost-utility methods to conduct economic analysis of these three periodontal treatments.

## Materials and methods

### Study design

**Fig 1:**
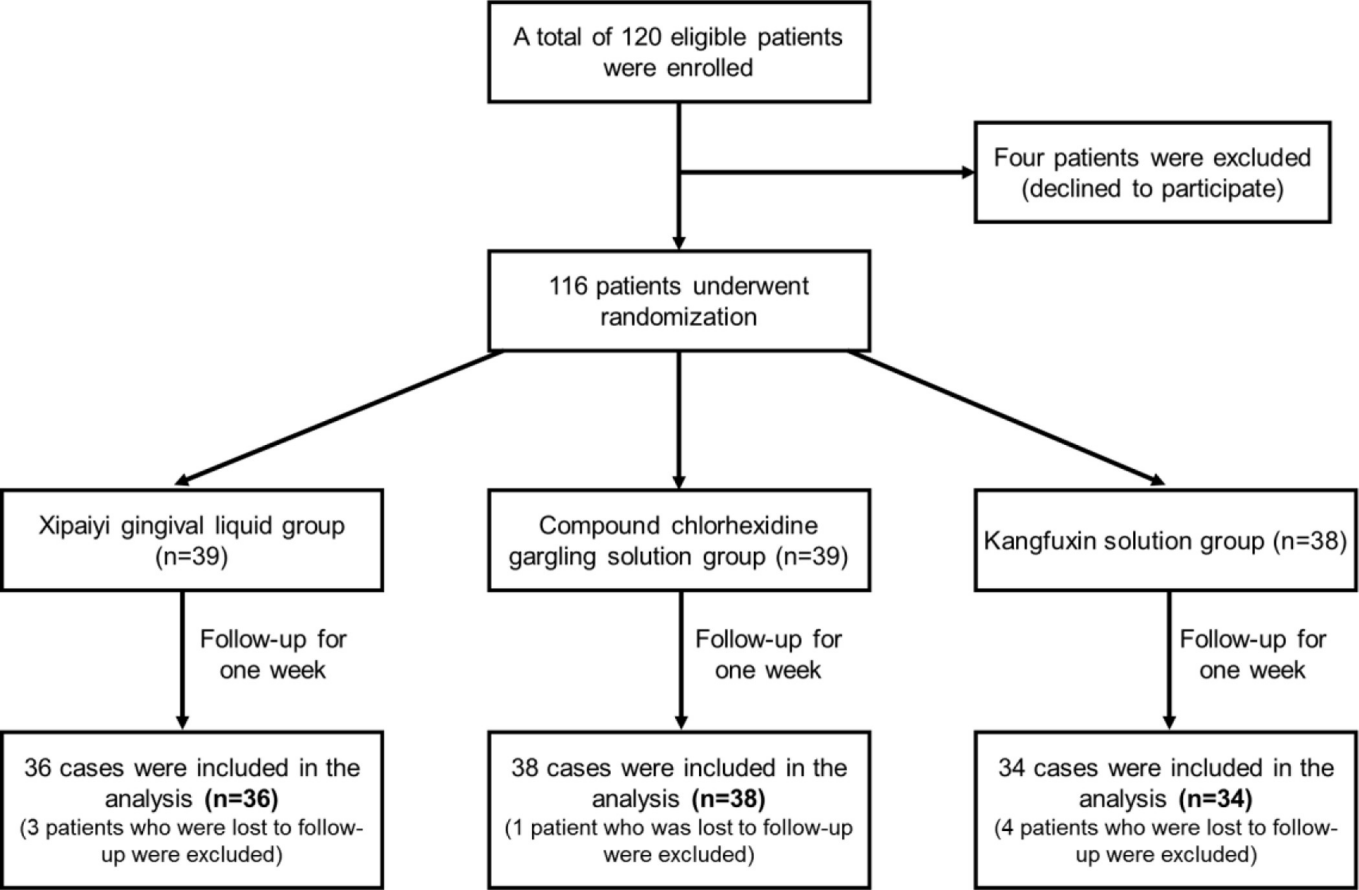
Flow chart of the inclusion criteria and exclusion criteria for patients with chronic periodontitis.

A total of 108 patients with chronic periodontitis admitted to the Department of Periodontology, Stomatological Hospital, Xuzhou Medical between January 2022 and November 2022 were selected and randomly divided into one of the following three groups: group A, Xipaiyi gingival liquid group, *n* = 36; group B, compound chlorhexidine gargling solution group, *n* = 38, and group C, Kangfuxin solution group, *n* = 34 (Fig 1).

The inclusion criteria for the participants in this study were as follows: aged ≥18 years old; met the diagnostic criteria of chronic periodontitis; periodontal redness and swelling, bleeding on probing, and periodontal depth of more than 3 mm; the number of teeth was ≥20, and the number of teeth was ≥5 in each region; had not received periodontal treatment within 1 year, and had not used antibiotics or nonsteroidal anti-inflammatory drugs in the past 2 months; and timely return visit and complete follow-up.

Subjects were excluded from participating in this study if any of the following conditions were met: hypertension or diabetes; serious diseases of other organs, including malignant tumors, severe liver or kidney failure, or respiratory failure; pregnant or lactating women; allergy to the study drugs; and mental illness.

This study was approved by the Ethics Committee of the Xuzhou Stomatological Hospital (No: 2021–021). Due to the retrospective nature of this study, informed consent (written/verbal) of the patients was waived.

### Therapeutic method

All three groups received basic periodontal treatment (oral hygiene education, periodontal cleaning, ultrasonic supragingival and subgingival scaling, and dental surface smoothing). All patients also received one of the following gargles (10 mL for 5 min after three meals/day for 1 week): Xipaiyi gingival fixing solution (group A), compound chlorhexidine gargle (group B), or Kangfuxin solution (group C).

### Evaluation index and evaluation method

#### Cost index

Due to the lack of a unified method for calculating indirect costs and invisible costs as well as to avoid data bias, indirect costs and invisible costs were ignored, and only direct costs were considered. The direct costs included drug costs (the total cost of drugs during treatment and the cost of drugs for the treatment of oral complications), treatment costs (the cost of basic treatment for periodontitis scaling and the cost of treatment for adverse reactions), examination costs (the cost of examination during the patient’s treatment), and hospitalization costs (the cost of hospitalization for the patient’s adverse reactions).

#### Effectiveness index

The total effective rate of clinical treatment was used as the effectiveness index, and the clinical efficacy was evaluated according to the Guidelines for the Diagnosis and Treatment of Oral Diseases as follows: significant effect, the depth of the periodontal pocket was shortened by more than 3 mm, the periodontal attachment loss was decreased by more than 50%, the degree of tooth mobility was reduced by I–II degrees, and the symptoms of gingival pain and redness had completely disappeared; effective, the depth of the periodontal pocket was significantly shortened, periodontal attachment loss was significantly decreased, tooth loosening was significantly relieved, gum pain completely disappeared, and redness and swelling symptoms basically disappeared; and no effect, there was no remarkable change in the depth of the periodontal pocket, periodontal attachment loss, tooth mobility and gingival pain, or redness. Total effective rate = (markedly effective cases+effective cases)/total cases×100%.

#### Utility index

Quality-adjusted life years (QALYs) were used as the utility index, QALYs = reported survival time×health utility value. In this study, the Chinese version of the 3-level version of the EuroQol Five Dimensions Questionnaire (EQ-5D-3L) was used to measure the utility value of the quality of life of patients with periodontitis before and after the rehabilitation treatment. The EQ-5D-3L Chinese effect value scoring system, established by Gordon Liu *et al*., was used to convert the health utility value of patients with periodontitis [16]. For example, the utility value of “13212,” calculated by China’s utility scoring system, is U = 1- (0.039+0.208+0.074+0.086+0.022) = 0.571. If the five dimension levels are all equal to 1, the EQ-5D utility value is 1; if the five dimension levels are all equal to 0, the EQ-5D utility value is -0.149. Therefore, the EQ-5D utility value range is [-0.149, 1].

#### Evaluation methodology

In this study, the cost of periodontitis treatment, the effective rate, and QALYs before and after rehabilitation treatment were collected, and the cost-effectiveness ratio (CER) and cost-utility ratio (CUR) were used to present the economic analysis results. According to the Guidelines for Pharmacoeconomic Evaluation in China (2020), when the CER and CUR are less than the gross domestic product (GDP) per capita, the cost of the treatment plan is completely worthwhile [17].

### Statistical analysis

SPSS25.0 was used for statistical analysis. The qualitative data were expressed as the rate [*n* (%)], and the quantitative data were expressed as the mean ± standard deviation.

## Results

### Patient characteristics

A total of 108 patients with chronic periodontitis admitted to the Department of Periodontology, Stomatological Hospital, Xuzhou Medical University from January 2022 to November 2022 were randomly divided into the following three experimental groups: group A, 23 males and 13 females, mean age of 42.06±9.33 years old (range: 25–61 years old); group B, 22 males and 16 females, mean age of 45.24±11.57 years old (range 19–78 years old); group C, 15 males and 19 females, mean age of 48.71±15.52 years old (range: 23–69 years old). There were no significant differences in baseline data such as sex, age, smoking habits, and drinking habits among the three groups (Table 1; *P*>0.05).

**Table 1:** Comparison of basic data among the three groups.

Parameter	Group A *N* = 36	Group B *N* = 38	Group C *N* = 34	*P*-value
Sex, *n* (% male)	23 (63.89)	22 (57.89)	15 (44.12)	0.235
Age, years	42.06±9.33	45.24±11.57	48.71±15.52	0.426
Smokers, *n* (%)	11 (30.56)	11 (28.95)	10 (29.41)	0.988
Drinkers, *n* (%)	6 (16.67)	7 (18.42)	5 (14.71)	0.915

### Changes of periodontal parameters before and after treatment

There were no significant differences in the periodontal pocket depth (mm), periodontal attachment loss (mm), plaque index (PLI, points), or sulcus bleeding index (SBI, points) among the three groups before treatment (*P*>0.05). After treatment, the periodontal indexes of the three groups were markedly decreased, and the PLI and SBI indexes of group B were observably better than those of group A and group C (S1 Table; *P*<0.05).

### Comparison of efficacy evaluations

The overall effective rates of the three groups were 83.33% in group A, 78.95% in group B, and 76.47% in group C. The effective rates of the three groups were statistically significant (Table 2; *H* = 6.462, *P* = 0.040).

**Table 2:** Comparison of efficacy evaluation among the three groups [*n* (%)].

Group	Inefficient	Effective rate	Excellent effective rate	Overall effective rate
Group A	6 (16.67)	9 (25.0)	21 (58.33)	30 (83.33)
Group B	8 (21.05)	17 (44.73)	13 (34.21)	30 (78.95)
Group C	8 (23.53)	18 (52.94)	8 (23.53)	26 (76.47)
*H*-value				6.462
*P*-value				0.040

### Comparison of treatment costs

The average costs of the gargles in the three experimental groups were 231.62 RMB (group A), 129.78 RMB (group B), and 164.92 RMB (group C), and the differences were statistically significant (*F* = 8.033, *P*<0.05). The average total costs of the three groups were 1523.9 RMB, 1242.15 RMB, and 1435.28 RMB, respectively. There was a prominent difference in the average total cost among the three groups (Tables 3 and 4; *F* = 4.705, *P*<0.05).

**Table 3:** Cost structure distribution.

Group	Unit cost	Dosage regimen	Number of cycles per 7 days	Price per period	Source of price
Group A	¥33.08/30 mL	30 mL/day	1	¥231.62	local prices
Group B	¥55.62/90 mL	30 mL/day	1	¥129.78	local prices
Group C	¥94.24/120 mL	30 mL/day	1	¥164.92	local prices

**Table 4:** Costs of treatment options for the three groups.

Group	Gargle cost (RMB)	Cost of primary periodontal therapy (RMB)	Average total cost (RMB)
Group A	231.62	1292.29	1523.9
Group B	129.78	1112.38	1242.15
Group C	164.92	1269.86	1435.28
*F*-value	8.033	2.469	4.705
*P*-value	0.001	0.09	0.011

### Cost analysis of periodontitis treatment in the three groups

#### Cost-effectiveness analysis

The CER was calculated according to the total cost (C)/effective rate (E). The CERs of groups A, B, and C were 1828.75 RMB, 1573.34 RMB, and 1876.92 RMB, respectively (Table 5).

**Table 5:** Cost-effectiveness analysis of rehabilitation for the three groups of patients with periodontitis.

Group	Total cost (RMB)	Effective rate	Cost/effectiveness (RMB)
Group A	1523.9	83.33%	1828.75
Group B	1242.15	78.95%	1573.34
Group C	1435.28	76.47%	1876.92

#### Cost-utility analysis

The EQ-5D-3L scale was used as the utility value for the treatment effect of periodontitis in the three groups, and the QALYs were calculated. According to the “Jiangsu Province Aging Development Report (2022),” the average life expectancy of the population in Jiangsu Province is 79.32 years old. According to the formula for QALYs, the QALYs of the three groups were calculated. The patients needed 213.43 RMB (group A), 195.61 RMB (group B), and 301.53 RMB (group C) for each QALY (Table 6).

**Table 6:** Cost-utility analysis of rehabilitation for the three groups of patients with periodontitis.

Group	Pre-treatment utility value	Post-treatment utility value	Acquired utility value	QALYs	CUR (C/QALYs)
Group A	0.92	0.98	0.09	7.14	213.43 RMB
Group B	0.90	0.98	0.08	6.35	195.61 RMB
Group C	0.91	0.97	0.06	4.76	301.53 RMB

QALYs, quality-adjusted life years; CUR, cost-utility ratio.

### Sensitivity analysis

#### Univariate sensitivity analysis

The uncertainty of the pharmacoeconomic data will affect the results to a certain extent. Therefore, sensitivity analysis was used to test the influence of each variable on the results when they fluctuated within a certain range. According to the related policies of centralized drug procurement, it is inevitable that the drug costs will be reduced and the diagnosis and treatment costs will be increased. The CERs and CURs were calculated for each group (Table 7), assuming a 10% decrease in the price of mouthwash and a 10% increase in the cost of care. The CERs were 1956.05 RMB (group A), 1697.81 RMB (group B), and 2020.76 RMB (group C); while the CURs were 228.29 RMB (group A), 211.09 RMB (group B), and 324.64 RMB (group C). These findings indicated that group B had the lowest values, so the reduction of drug costs and the increase of diagnosis and treatment costs did not affect the analysis results.

**Table 7:** Cost-effectiveness and cost-utility sensitivity analyses of rehabilitation for periodontitis patients in the three groups.

Group	Total cost (C*)	CER (C*/E)	CUR (C*/QALYs)
Group A	1629.98 RMB	1956.05 RMB	228.29 RMB
Group B	1340.42 RMB	1697.81 RMB	211.09 RMB
Group C	1545.27 RMB	2020.76 RMB	324.64 RMB

CER, cost-effectiveness ratio; CUR, cost-utility ratio.

### Probabilistic sensitivity analysis

**Fig 2:**
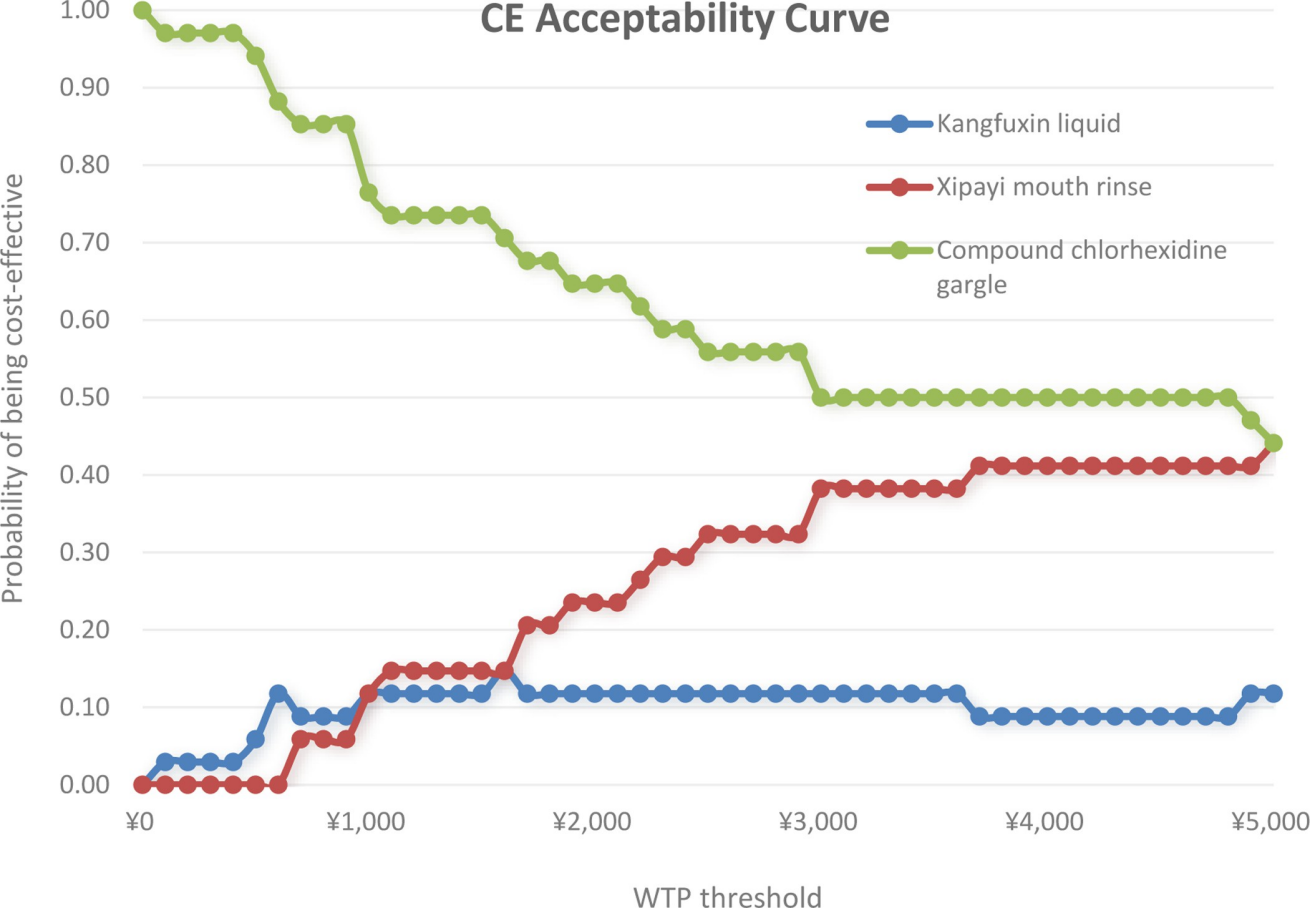
Cost-effectiveness acceptability curve of Kangfuxin liquid vs. Xipayi gingival liquid vs. compound chlorhexidine gargle.

We conducted bootstrapping on the results of the study, and the statistical analysis showed that the compound chlorhexidine gargle regimen was the absolute dominant regimen when it was less than 5000 yuan. According to the Guidelines for Pharmacoeconomic Evaluation in China, the willingness-to-pay threshold was set at 1–3 times China’s GDP per capita. However, the GDP per capita reached 89.4 thousand yuan, which was far beyond the cost of this treatment regimen, so 1–3 times the cost of this treatment regimen was used for sensitivity analysis. In this study, the maximum treatment cost was 1500 yuan (RMB). When the willingness-to-pay threshold was 1500 yuan /QALY, the probability of the compound chlorhexidine gargle, Xipayi gingival liquid, and Kangfuxin solution being economical was 75%, 15%, and 11%, respectively (Fig 2). It can be seen that the compound chlorhexidine gargle protocol is economical under the willingness-to-pay threshold set in this study.

## Discussion

The CEA and CUA methods were used to study the economic effects of three mouthwashes in the treatment of periodontitis. The results showed that the effective rates of the three mouthwashes were 83.33%, 78.95%, and 76.47%, respectively (*H* = 6.462, *P* = 0.040), and there was a significant difference among the three groups in the effective rates (*P*<0.05). The PLI scores (*P*<0.05) and SBI scores (*P*<0.05) indicated that the three gargles had significant effects on inhibiting plaque and improving gingival bleeding in patients with periodontitis.

Chronic periodontitis is a chronic infectious disease caused by a variety of bacteria. Plaque microorganisms initiate periodontal disease. Therefore, in clinical treatment, dental plaque and calculus are usually cleaned first to eliminate pathogenic factors. Basic periodontal treatments such as supragingival and subgingival scaling have good clinical efficacy and have been widely used in clinical practice [18]. In order to better control inflammation and reduce recurrence, a gargle is usually used in conjunction with treatment. The main component of Kangfuxin liquid is the extract of *Americana periplanella*, which contains a variety of active substances such as polyols, mucin, epidermal growth factor, and human essential amino acids. It has been widely used to treat oral diseases, such as reducing the inflammatory response and improving microcirculation [19]. In addition, the traditional Chinese medicine preparation composed of the gallic extract of Xipayi gingival liquid has the functions of strengthening teeth, strengthening gingiva, clearing blood, and relieving pain. Some studies have shown that Xipayi gingival liquid has a significant effect on periodontitis and can effectively reduce the indexes of periodontitis [20]. Meanwhile, compound chlorhexidine gargle is a broad-spectrum microbicide, with chlorhexidine gluconate and metronidazole as the main ingredients. Chlorhexidine gluconate is adsorbed in the permeability barrier of the bacterial cytoplasmic membrane; therefore, it is responsible for leaking out cell contents, playing an antibacterial role, reducing dental plaque accumulation and the bleeding index, and improving oral health [21]. The three types of gargles had significant effects on improving periodontitis and could effectively reduce the inflammatory indicators of periodontitis. However, the costs of the three mouthwashes used in clinical practice were quite different. Therefore, this study conducted an economic analysis of the three periodontal treatments.

In this study, the direct cost was used for the pharmacoeconomic evaluation, including the cost of the gargle and the cost of the initial periodontal treatment. Ultrasonic supragingival scaling plus ultrasonic subgingival scaling plus dental surface planing was used for the initial periodontal treatment of the enrolled patients, so there was no significant difference in the cost of the periodontal treatment among the three groups (*F* = 2.469, *P* = 0.09). The average costs of the three gargles were 231.62 RMB, 129.78 RMB, and 164.92 RMB, respectively (*F* = 8.033, *P* = 0.01), and the total costs were 1523.9 RMB, 1242.15 RMB, and 1435.28 RMB, respectively (*F* = 4.705, *P*<0.05); thus, the results were significantly different.

The CERs of the three groups were 1828.75 RMB, 1573.34 RMB, and 1876.92 RMB, respectively, and the per capita GDP of Jiangsu Province in 2022 was 144,400 RMB [22]. The CERs of the three groups were less than the per capita GDP. According to the current average economic income and living standard of the Chinese population, the findings of this study indicated that the treatment costs of the three groups of patients were completely worth it. Among them, the CER of the compound chlorhexidine group was the lowest, indicating that group B spent the lowest cost to achieve the same treatment effect.

In this study, the Chinese version of EQ-5D-3L and the China effect value scoring system were used to convert the health utility values of patients with periodontitis, and the QALYs of the three groups were calculated. The results revealed that each QALY obtained by the patients cost 213.43 RMB, 195.61 RMB, and 301.53 RMB, respectively, which were less than the per capita GDP of Jiangsu Province, indicating that the treatment was completely worth it. The cost of the compound chlorhexidine group was the lowest per complete QALY obtained, which was consistent with the results of the cost-effectiveness analysis.

In order to alleviate the burden of high drug prices, China has controlled drug prices. China has pushed forward the reform of its medical system and implemented a centralized drug procurement system in medical institutions in all provinces, cities, and regions. Through centralized procurement of drugs for routine use and drugs with high consumption, the drug procurement process has been standardized and drug procurement prices have been effectively reduced. With the widespread application of pharmacoeconomics in drug evaluation, economic analysis of three kinds of gargles in the treatment of periodontitis can more intuitively find the differences among them, thus providing reference for the inclusion of a certain gargle in the centralized procurement list.

According to the guidelines for the Management of Comprehensive Clinical Evaluation of Drugs in China and the requirements of the comprehensive clinical evaluation of drugs in Jiangsu Province, this study is in line with the “small evaluation” work of the hospital, and the results can be used as a reference for the comprehensive clinical evaluation of drugs.

However, this study had certain limitations that must be addressed. First, the sample size was small, and only the direct cost was considered, which may have caused a certain bias in the study results. Second, a consistent assumption was made for other costs, and the effect of separate changes in the prices of other drugs was not considered. Finally, the determination of variables and their variation ranges in the sensitivity analysis were subjective and lacked guidance or standard support; therefore, they may have led to potential bias. Various factors should be considered to make the results more convincing in a follow-up study.

## Conclusion

In conclusion, this study used both cost-effectiveness and cost-utility analyses to evaluate the cost-effectiveness of various mouthwashes in the treatment of periodontitis. The results indicated that the cost of mouthwash treatment was acceptable and worthwhile for patients to increase the treatment efficiency and to gain a full QALY, and compound chlorhexidine was the least expensive mouthwash. Sensitivity analysis verified the influence of variables on the results when they fluctuated within a certain range. Under the influence of centralized drug procurement related policies, a 10% reduction in drug cost and a 10% increase in diagnosis and treatment cost had no effect on the results. The compound chlorhexidine group was still the group with the lowest CER and CUR.

## Supporting information

S1 Table Comparison of periodontal indexes before and after treatment among the three groups. (DOCX)

S1 Checklist STROBE statement—checklist of items that should be included in reports of observational studies. (DOCX)
