# Supplementary material for: Cost-effectiveness and cost-utility analyses of three different gargles in the treatment of chronic periodontitis
Source: PLoS One. 2024 May 8;19(5):e0302592. doi: 10.1371/journal.pone.0302592 (PMC11078434; doi:10.1371/journal.pone.0302592)
Supplement: S1 Table — (DOCX) [file pone.0302592.s001.docx]

**Table S1** Comparison of periodontal indexes before and after treatment among the three groups

| Group | Cases | PD (mm) | |  | AL (mm) | |  | PLI (score) | |  | SBI (score) | |
| --- | --- | --- | --- | --- | --- | --- | --- | --- | --- | --- | --- | --- |
|  |  | Pre-treatment | Post-treatment |  | Pre-treatment | Post-treatment |  | Pre-treatment | Post-treatment |  | Pre-treatment | Post-treatment |
| Group A | 36 | 4.58±0.87 | 2.57±0.97 |  | 2.24±1.05 | 1.04±1.03 |  | 3.00±0.56 | 1.22±0.79 |  | 3.21±0.66 | 1.51±0.82 |
| Group B | 38 | 4.45±0.54 | 2.47±0.73 |  | 2.47±0.74 | 1.46±1.11 |  | 2.83±0.60 | 0.97±0.40 |  | 3.13±0.70 | 0.88±0.44 |
| Group C | 34 | 5.12±1.05 | 2.93±0.99 |  | 2.51±0.69 | 0.94±0.87 |  | 2.97±0.61 | 1.44±0.68 |  | 3.62±0.56 | 2.38±1.04 |
| *F*-value |  | 1.726 | 2.475 |  | 1.701 | 2.723 |  | 0.204 | 4.802 |  | 1.702 | 32.023 |
| *P*-value |  | 0.086 | 0.089 |  | 0.107 | 0.07 |  | 0.960 | 0.01 |  | 0.128 | 0 |

PD, periodontal pocket depth; AL, periodontal attachment loss; PLI, plaque index; SBI, sulcus bleeding index.
