# Supplementary material for: Cost-effectiveness and cost-utility analyses of three different gargles in the treatment of chronic periodontitis
Source: PLoS One. 2024 May 8;19(5):e0302592. doi: 10.1371/journal.pone.0302592 (PMC11078434; doi:10.1371/journal.pone.0302592)
Supplement: S1 Checklist — (DOCX) [file pone.0302592.s002.docx]

STROBE Statement—checklist of items that should be included in reports of observational studies

|  | Item No. | Recommendation | Page  No. | Relevant text from manuscript |
| --- | --- | --- | --- | --- |
| **Title and abstract** | 1 | (*a*) Indicate the study’s design with a commonly used term in the title or the abstract | 2 | This study aimed to investigate the economics of three different gargles in the treatment of chronic periodontitis. |
|  |  | (*b*) Provide in the abstract an informative and balanced summary of what was done and what was found | 2 | The CER and CUR results were the same, and the compound chlorhexidine group was the lowest, demonstrating that when the same therapeutic effect was achieved, it cost the least. |
| Introduction | | | |  |
| Background/rationale | 2 | Explain the scientific background and rationale for the investigation being reported | 4 | However, there are few economic studies on periodontitis treatments, and in those that have been conducted, their effectiveness is often limited to a single country and its social and economic situation, and some studies only estimated the time required to perform various periodontal treatment procedures without evaluating the economics of their treatment |
| Objectives | 3 | State specific objectives, including any prespecified hypotheses | 4 | Therefore, in this study, we used cost-effectiveness and cost-utility methods to conduct economic analysis of these three periodontal treatments. |
| Methods | | | |  |
| Study design | 4 | Present key elements of study design early in the paper | 4 | A total of 108 patients with chronic periodontitis were selected and randomly divided into one of the following three groups |
| Setting | 5 | Describe the setting, locations, and relevant dates, including periods of recruitment, exposure, follow-up, and data collection | 4-5 | 108 patients with chronic periodontitis admitted to the Department of Periodontology, Stomatological Hospital, Xuzhou Medical between January 2022 and November 2022 |
| Participants | 6 | (*a*) *Cohort study*—Give the eligibility criteria, and the sources and methods of selection of participants. Describe methods of follow-up  *Case-control study*—Give the eligibility criteria, and the sources and methods of case ascertainment and control selection. Give the rationale for the choice of cases and controls  *Cross-sectional study*—Give the eligibility criteria, and the sources and methods of selection of participants | 5 | The inclusion criteria of the participants in this study were as follows |
|  |  | (*b*) *Cohort study*—For matched studies, give matching criteria and number of exposed and unexposed  *Case-control study*—For matched studies, give matching criteria and the number of controls per case | 5 | Subjects were excluded from participating in this study if any of the following conditions were met |
| Variables | 7 | Clearly define all outcomes, exposures, predictors, potential confounders, and effect modifiers. Give diagnostic criteria, if applicable | 5 | All three groups received basic periodontal treatment (oral hygiene education, periodontal cleaning, ultrasonic supragingival and subgingival scaling, and dental surface smoothing) |
| Data sources/ measurement | 8* | For each variable of interest, give sources of data and details of methods of assessment (measurement). Describe comparability of assessment methods if there is more than one group | *6-7* | Due to the lack of a unified method for calculating indirect costs and invisible costs as well as to avoid data bias, indirect costs and invisible costs were ignored, and only direct costs were considered. |
| Bias | 9 | Describe any efforts to address potential sources of bias | 6-7 | Due to the lack of a unified method for calculating indirect costs and invisible costs as well as to avoid data bias, indirect costs and invisible costs were ignored, and only direct costs were considered. |
| Study size | 10 | Explain how the study size was arrived at |  |  |

Continued on next page

| Quantitative variables | 11 | Explain how quantitative variables were handled in the analyses. If applicable, describe which groupings were chosen and why | 6-7 | In this study, the cost of periodontitis treatment, the effective rate, and QALYs before and after rehabilitation treatment were collected, and the cost-effectiveness ratio (CER) and cost-utility ratio (CUR) were used to present the economic analysis results. |
| --- | --- | --- | --- | --- |
| Statistical methods | 12 | (*a*) Describe all statistical methods, including those used to control for confounding | 7 | SPSS25.0 was used for statistical analysis. The qualitative data were expressed as the rate [*n* (%)], and the quantitative data were expressed as the mean ± standard deviation. |
|  |  | (*b*) Describe any methods used to examine subgroups and interactions | 7 | SPSS25.0 was used for statistical analysis. The qualitative data were expressed as the rate [*n* (%)], and the quantitative data were expressed as the mean ± standard deviation. |
|  |  | (*c*) Explain how missing data were addressed | 7 | SPSS25.0 was used for statistical analysis. The qualitative data were expressed as the rate [*n* (%)], and the quantitative data were expressed as the mean ± standard deviation. |
|  |  | (*d*) *Cohort study*—If applicable, explain how loss to follow-up was addressed  *Case-control study*—If applicable, explain how matching of cases and controls was addressed  *Cross-sectional study*—If applicable, describe analytical methods taking account of sampling strategy | 7 | SPSS25.0 was used for statistical analysis. The qualitative data were expressed as the rate [*n* (%)], and the quantitative data were expressed as the mean ± standard deviation. |
|  |  | (*e*) Describe any sensitivity analyses | 7 | SPSS25.0 was used for statistical analysis. The qualitative data were expressed as the rate [*n* (%)], and the quantitative data were expressed as the mean ± standard deviation. |
| Results | | | | |
| Participants | 13* | (a) Report numbers of individuals at each stage of study—eg numbers potentially eligible, examined for eligibility, confirmed eligible, included in the study, completing follow-up, and analysed | 8 | A total of 108 patients with chronic periodontitis admitted to the Department of Periodontology, Stomatological Hospital, Xuzhou Medical University from January 2022 to November 2022 were randomly divided into the following three experimental groups |
|  |  | (b) Give reasons for non-participation at each stage | 8 | There were no significant differences in baseline data such as sex, age, smoking habits, and drinking habits among the three groups |
|  |  | (c) Consider use of a flow diagram |  |  |
| Descriptive data | 14* | (a) Give characteristics of study participants (eg demographic, clinical, social) and information on exposures and potential confounders | 8 | group A, 23 males and 13 females, mean age of 42.06±9.33 years old (range: 25–61 years old); group B, 22 males and 16 females, mean age of 45.24±11.57 years old (range 19–78 years old); group C, 15 males and 19 females, mean age of 48.71±15.52 years old (range: 23–69 years old). |
|  |  | (b) Indicate number of participants with missing data for each variable of interest | 8 | group A, 23 males and 13 females, mean age of 42.06±9.33 years old (range: 25–61 years old); group B, 22 males and 16 females, mean age of 45.24±11.57 years old (range 19–78 years old); group C, 15 males and 19 females, mean age of 48.71±15.52 years old (range: 23–69 years old). |
|  |  | (c) *Cohort study*—Summarise follow-up time (eg, average and total amount) | 8 | from January 2022 to November 2022 |
| Outcome data | 15* | *Cohort study*—Report numbers of outcome events or summary measures over time | *8* | There were no significant differences in the periodontal pocket depth (mm), periodontal attachment loss (mm), plaque index (PLI, points), or sulcus bleeding index (SBI, points) among the three groups before treatment |
|  |  | *Case-control study—*Report numbers in each exposure category, or summary measures of exposure |  |  |
|  |  | *Cross-sectional study—*Report numbers of outcome events or summary measures |  |  |
| Main results | 16 | (*a*) Give unadjusted estimates and, if applicable, confounder-adjusted estimates and their precision (eg, 95% confidence interval). Make clear which confounders were adjusted for and why they were included | 9 | The overall effective rates of the three groups were 83.33% in group A, 78.95% in group B, and 76.47% in group C. The effective rates of the three groups were statistically significant (Table 2; *H*=6.462, *P*=0.040). |
|  |  | (*b*) Report category boundaries when continuous variables were categorized |  |  |
|  |  | (*c*) If relevant, consider translating estimates of relative risk into absolute risk for a meaningful time period |  |  |

Continued on next page

| Other analyses | 17 | Report other analyses done—eg analyses of subgroups and interactions, and sensitivity analyses | 11 | Therefore, sensitivity analysis was used to test the influence of each variable on the results when they fluctuated within a certain range. |
| --- | --- | --- | --- | --- |
| Discussion | | | | |
| Key results | 18 | Summarise key results with reference to study objectives | 12-13 | The results showed that the effective rates of the three mouthwashes were 83.33%, 78.95%, and 76.47%, respectively (*H*=6.462, *P*=0.040), and there was a significant difference among the three groups in the effective rates (*P*<0.05). |
| Limitations | 19 | Discuss limitations of the study, taking into account sources of potential bias or imprecision. Discuss both direction and magnitude of any potential bias | 13 | In this study, the direct cost was used for the pharmacoeconomic evaluation, including the cost of the gargle and the cost of the initial periodontal treatment. |
| Interpretation | 20 | Give a cautious overall interpretation of results considering objectives, limitations, multiplicity of analyses, results from similar studies, and other relevant evidence | 13-14 | Ultrasonic supragingival scaling plus ultrasonic subgingival scaling plus dental surface planing was used for the initial periodontal treatment of the enrolled patients, so there was no significant difference in the cost of the periodontal treatment among the three groups |
| Generalisability | 21 | Discuss the generalisability (external validity) of the study results | 15 | However, this study had certain limitations that must be addressed. First, the sample size was small, and only the direct cost was considered, which may have caused a certain bias in the study results. |
| Other information | |  | | |
| Funding | 22 | Give the source of funding and the role of the funders for the present study and, if applicable, for the original study on which the present article is based | 15 | This work was supported by the Tianqing Hospital Pharmaceutical Fund Research Project of Jiangsu Pharmaceutical Society [grant numbers Q202141] and Pharmaceutical Research Project of Xuzhou Municipal Health Commission [grant numbers XWYXKY202109]. |

*Give information separately for cases and controls in case-control studies and, if applicable, for exposed and unexposed groups in cohort and cross-sectional studies.

**Note:** An Explanation and Elaboration article discusses each checklist item and gives methodological background and published examples of transparent reporting. The STROBE checklist is best used in conjunction with this article (freely available on the Web sites of PLoS Medicine at http://www.plosmedicine.org/, Annals of Internal Medicine at http://www.annals.org/, and Epidemiology at http://www.epidem.com/). Information on the STROBE Initiative is available at www.strobe-statement.org.
